# Supplementary material for: pH as a Key Factor for the Quality Assurance of the Preparation of Gastrodiae Rhizoma Formula Granules
Source: Molecules. 2022 Nov 21;27(22):8091. doi: 10.3390/molecules27228091 (PMC9699468; doi:10.3390/molecules27228091)
Supplement: Supplementary file 1 [file molecules-27-08091-s001.zip › molecules-2011981-supplementary.pdf]

Supporting Information

For

pH as a key factor for the quality assurance of the preparation of Gastrodiae Rhizoma formula granules

Shuting Xie[1], Ke Min[1], Hai Li[1], Ying Wang[1], Mincong Liu[1], Ming Ma[1], Desheng Zhou[2,*], Haijun Tu[3,*], Bo Chen[1,*]

[1] Key Laboratory of Phytochemical R&D of Hunan Province and Key Laboratory of Chemical Biology & Traditional Chinese Medicine Research of Ministry of Education, Hunan Normal University, Changsha, 410081, China

[2] The First Affiliated Hospital of Hunan University of Chinese Medicine, Changsha 410000 China

[3] College of Biology, Hunan University, Changsha 410000, China

Contents

Table S1. The accuracy of the method was verified by adding 100% target compounds to samples.

Table S2. Investigation of the precision, repeatability and stability of six index components by HPLC.

Table S3. The pH of water extracts of eight raw materials in Huoxuerongluo Pill II.

Table S4. Sample information of *GR* decoction pieces.

Figure S1. The potential hydrolysis paths of parishin A.

Figure S2. HPLC chromatogram of the hydrolysis of parishin A at alkaline solution (pH = 10.30) with 10 min.

Figure S3. HPLC spectra of *GR* decoction piece and Huoxuerongluo Pill II.

Figure S4. Chemical structures of gastrodin, *p*-hydroxybenzyl alcohol, parishin A, parishin B, parishin C and parishin E in this study.

Table S1. The accuracy of the method was verified by adding 100% target compounds to samples. All calibration points were repeated three times (n = 3).

Six index components	Background content (mg/g)	Added (mg/g)	Recovery%	RSD%
gastrodin	1.58	1.58	98.8	1.6
p-hydroxybenzyl alcohol	0.10	0.10	101.6	2.3
parishin A	3.56	3.56	103.0	2.4
parishin B	1.99	1.99	99.0	1.2
parishin C	0.85	0.85	100.4	0.4
parishin E	4.27	4.27	96.5	1.9

Table S2. Investigation of the precision, repeatability and stability of six index components by HPLC.

Six index components	Precision (RSD, %)	Repeatability (RSD, %)	Stability (RSD, %)
gastrodin	0.65	1.71	1.53
p-hydroxybenzyl alcohol	0.90	1.03	1.22
parishin A	0.20	2.01	0.28
parishin B	1.45	1.54	1.33
parishin C	1.27	2.75	1.44
parishin E	0.51	2.75	1.49

Table S3. The pH of water extracts of eight raw materials in Huoxuerongluo Pill II.

Eight raw materials	pH
Lonicerae japonicae caulis	5.72
Picriae herba	4.86
GR	4.47
Chaenomelis fructus	3.37
Acori tatarinowii rhizoma	4.97
Persicae semen	6.16
Angelicae dahuricae radix	3.51
Paeoniae radix rubra	4.86

Note: The pH of Huoxuerongluo Pill II itself was 4.77.

Table S4. Sample information of *GR* decoction pieces.

No.	Batch number	Place of production	Production enterprise
S1	20081711	Hubei	Hunan Zhenxing Chinese Medicine Co.
S2	TH20113003	Hubei	The First Affiliated Hospital of Hunan University of Chinese Medicine
S3	20100802	Hubei	Hunan Zhenxing Chinese Medicine Co.
S4	191206	Hunan	Hunan Junhao Traditional Chinese Medicine trade Co.
S5	21031208	Hunan	Hunan Zhenxing Chinese Medicine Co.
S6	22032102	Hunan	Hunan Zhenxing Chinese Medicine Co.
S7	20210305	Sichuan	Hunan Chunzhibao Pharmaceutical Responsibility Co.
S8	D2203074	Sichuan	Sichuan Xin Lotus Traditional Chinese Medicine Co.
S9	20211008	Jilin	Hunan Yaoshengtang Chinese Medicine Technology Co.
S10	20201109	Jilin	Hunan Yaoshengtang Chinese Medicine Technology Co.
S11	2021030207	Jilin	Hunan Sanxiang Traditional Chinese Medicine Co.
S12	2201001	Yunnan	Hengtang Pharmaceutical Enterprise Co.
S13	2201002-1	Yunnan	Hengtang Pharmaceutical Enterprise Co.
S14	201810014	Yunnan	Yunnan Guohe Drug Co.

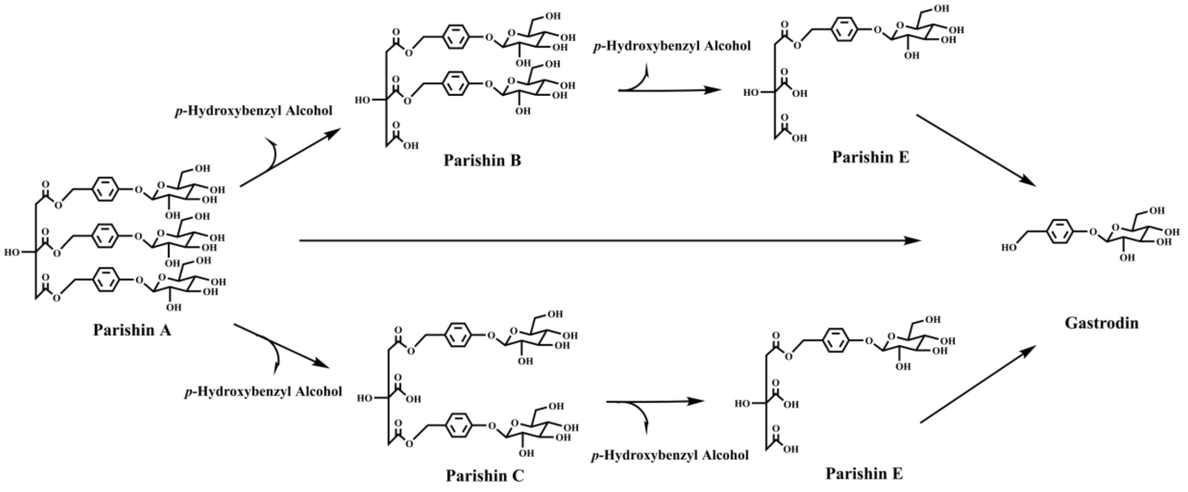

Figure S1. The potential hydrolysis paths of parishin A.

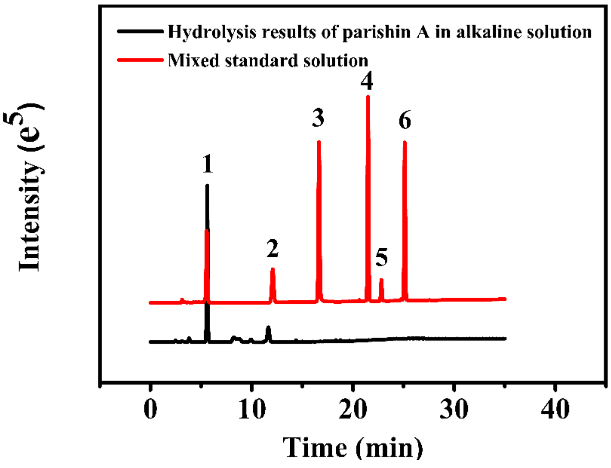

Figure S2. HPLC chromatogram of the hydrolysis of parishin A at alkaline solution (pH = 10.30) with 10 min. *Note:* Peak 1, 2, 3, 4, 5 and 6 refer to gastrodin, *p*-hydroxybenzyl alcohol, parishin E, parishin B, parishin C, and parishin A, respectively.

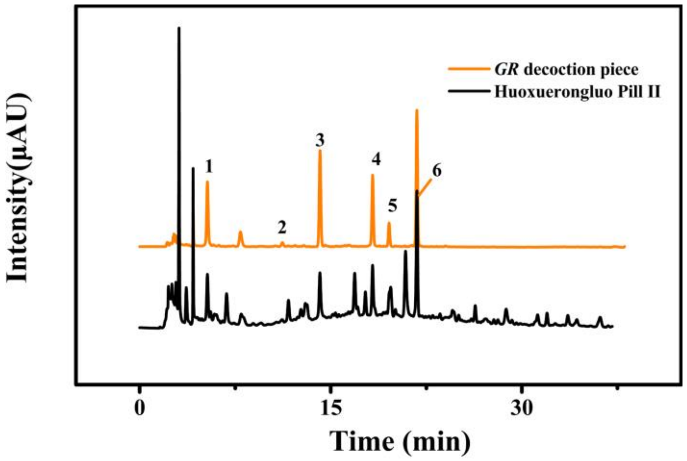

Figure S3. HPLC spectra of *GR* decoction piece and Huoxuerongluo Pill II. *Note:* Peak 1, 2, 3, 4, 5 and 6 refer to gastrodin, *p*-hydroxybenzyl alcohol, parishin E, parishin B, parishin C, and parishin A, respectively.

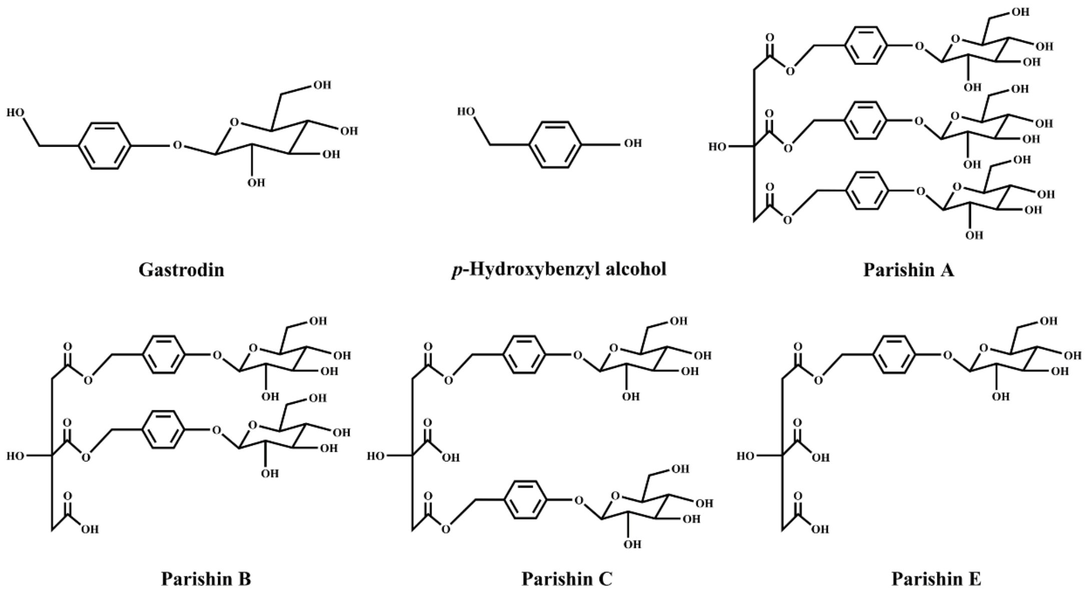

Figure S4. Chemical structures of gastrodin, *p*-hydroxybenzyl alcohol, parishin A, parishin B, parishin C and parishin E in this study.
